# Portal venous gas in intestinal malrotation with mild midgut volvulus

**DOI:** 10.1186/s40792-019-0700-z

**Published:** 2019-09-13

**Authors:** Ryuichiro Hirose, Hiroki Kai, Kaori Inatomi, Tsuyoshi Iwanaka, Naomi Morishima, Momotoshi Ikeda, Reiko Masaki, Akinori Iwasaki

**Affiliations:** 1 Department of General Thoracic, Breast, and Pediatric Surgery, Fukuoka University, 7-45-1, Nanakuma Jonan-ku, Fukuoka, 814-0180 Japan; 2 Division of Pediatrics, Fukuoka Central Hospital, 2-6-11, Yakuin Chuo-ku, Fukuoka, 810-0022 Japan; 3 Division of Radiological Technologist, Fukuoka Central Hospital, 2-6-11, Yakuin Chuo-ku, Fukuoka, 810-0022 Japan

**Keywords:** Portal venous gas, Malrotation, Midgut volvulus, Laparoscopic operation

## Abstract

**Background:**

Portal venous gas has been considered as a radiological sign requiring urgent operative intervention; however, the reports concerning portal venous gas associated with favorable outcome are recently increasing.

**Case presentation:**

We describe a 9-month-old boy with acute onset high fever and vomiting. The ultrasonography demonstrated micro-gas bubbles continuously floating in the intrahepatic portal vein. Contrast-enhanced CT, performed 1 h later from echography, revealed a whirlpool sign suggesting an intestinal malrotation with midgut volvulus, but with no signs of residual intrahepatic gas. Operative findings showed a mild volvulus with neither congestion nor ischemic change of the twisted bowel. Detorsion and Ladd’s procedure were completed laparoscopically.

**Conclusions:**

Transient portal venous gas bubbles may be generated even in the mild intestinal volvulus with no bowel ischemia. Ultrasonography can be a sensitive detector to visualize such small amounts of gas.

**Electronic supplementary material:**

The online version of this article (10.1186/s40792-019-0700-z) contains supplementary material, which is available to authorized users.

## Background

Portal venous gas (PVG) has been a well-known radiological finding, suggesting a potentially severe underlying abdominal disease requiring urgent operative intervention that occurs both in pediatric and adult populations [1]. Recently, spread of advanced imaging techniques has resulted in increased reports concerning PVG associated with not only severe or lethal conditions but also benign ones or diagnosed at much earlier stages [2]. The most reported cases concerning PVG with intestinal malrotation are associated with bowel necrosis induced by midgut volvulus [1, 3–7]. We describe a rare case of intestinal malrotation with mild volvulus who was diagnosed with air bubbles floating in the intrahepatic portal vein detected by bedside emergency ultrasonography.

## Case

A 9-month-old male infant weighing 8450 g presented to the primary care pediatrician with acute onset high fever, non-bilious vomiting, and continuous crying in a glum mood. He showed no bloody stool. On clinical examination, his abdomen was almost flat and it seemed that there is no apparent tenderness but he is crying constantly.

Blood investigations revealed no remarkable inflammation, and the white cell count was 7700/mm^3^ and CRP was 0.35 mg/dl (< 0.14) with normal coagulation parameters. Liver function tests showed mild transaminase elevations with ALT 57 U/L (10–42), AST 71 U/L (13–30), and normal level of total bilirubin 0.7 mg/dl (0.4–1.5). Blood urea was 3.5 mg/dl (< 20), and creatinine 0.26 mg/dl (< 1.07). Serum CK level was in the normal range with 237 U/L (59–248).

**Fig. 1:**
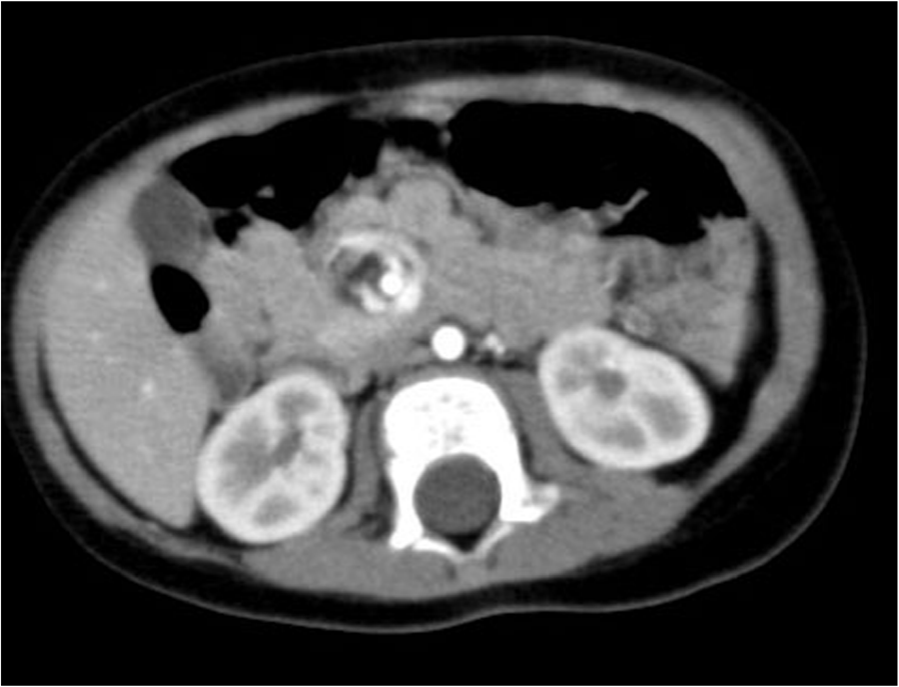
Contrast-enhanced CT: a whirlpool sign at the right upper abdomen. Neither intrahepatic portal venous gas nor signs of pneumatosis intestinalis were observed

**Fig. 2:**
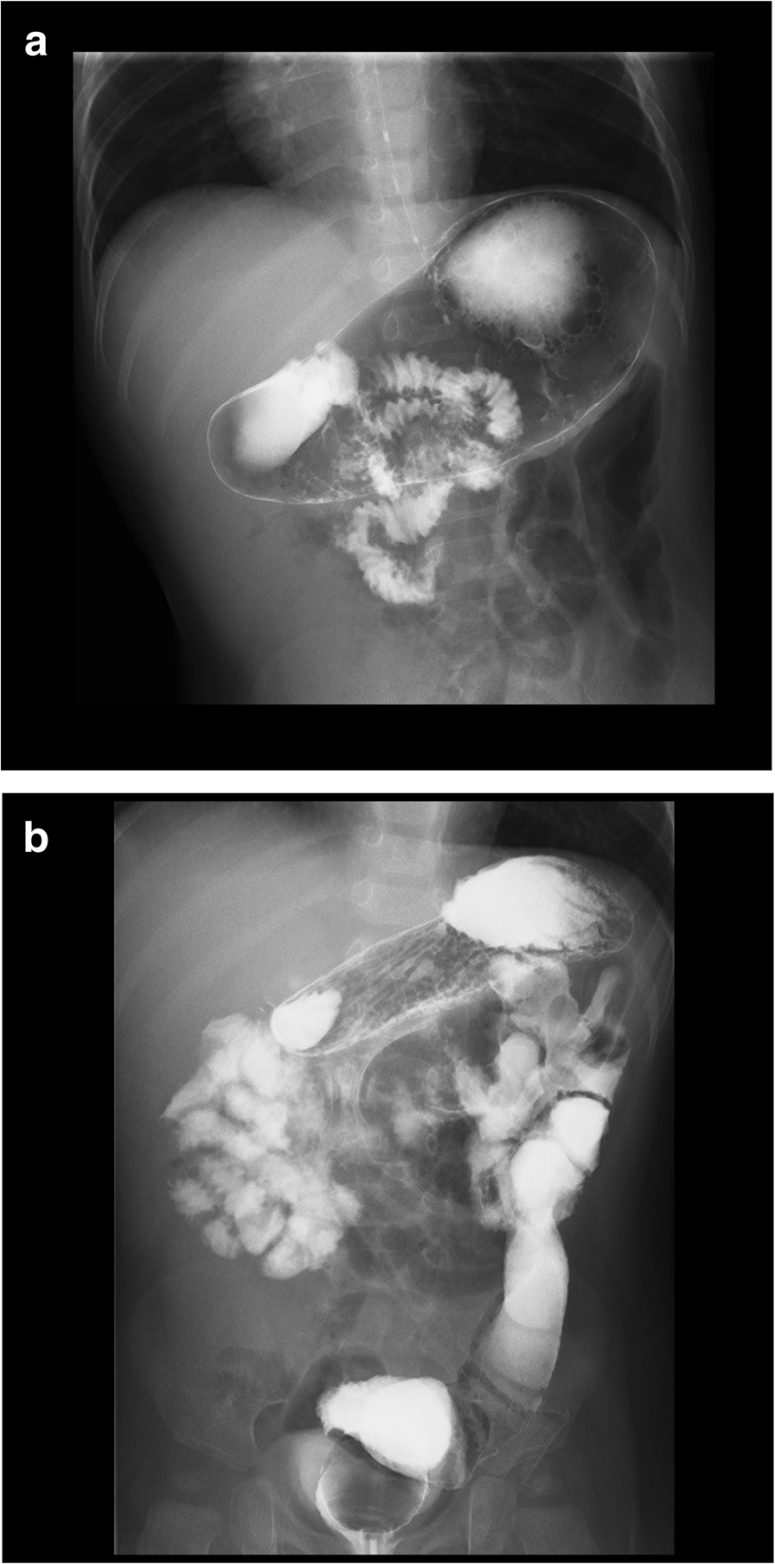
**a** Contrast upper gastrointestinal series showed a corkscrew sign of the jejunum. **b** Additional contrast enema showed a cecum at the mid-upper abdomen


The plain X ray-film showed no sign of bowel obstruction, but the ultrasound demonstrated micro-gas bubbles continuously floating in the intrahepatic portal vein, suggesting any deteriorating clinical problems (Additional file 1: Video S1). The infant was emergently transferred to our department. On admission, he appeared with no acute distress and showed no irritability. Contrast-enhanced CT, performed 1 h later from echography, revealed a whirlpool sign (Fig. 1) at the right upper abdomen but with neither intrahepatic portal venous gas nor signs of pneumatosis intestinalis (Fig. 2a). Contrast upper gastrointestinal series showed a corkscrew sign of the jejunum, and additional contrast enema showed a cecum at the mid-upper abdomen (Fig. 2b). The preoperative evaluation was concerned for intestinal malrotation with midgut volvulus.

**Additional file 1:** **Video S1.** Abdominal Ultrasound. The micro-bubbles of gas were detected as highly echogenic particles flowing within the intrahepatic portal vein. (MPG 7968 kb)


**Fig. 3:**
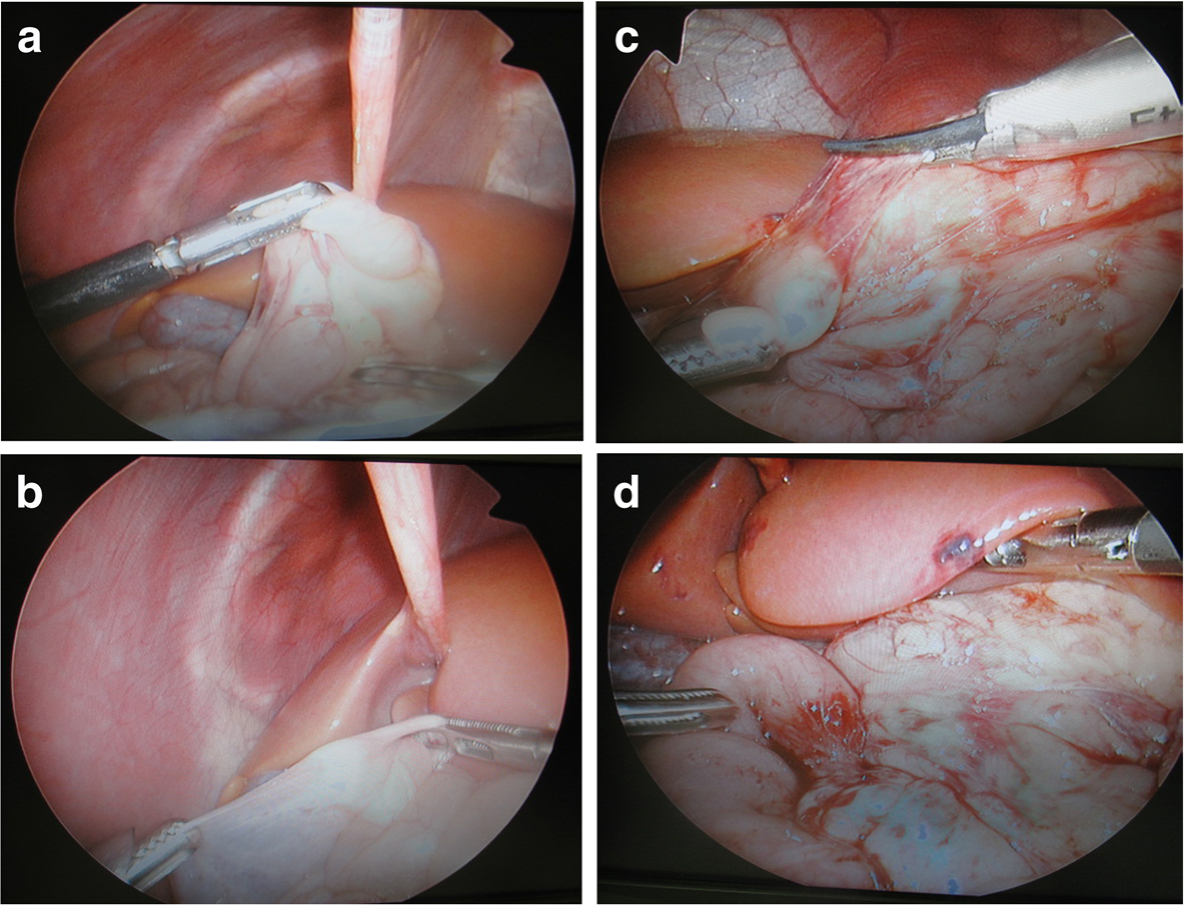
Operative view. **a** The cecum was located just under the liver at mid-abdomen. **b**, **c** The thick Ladd’s band was dissected using ultrasonic surgical device. **d** Mesentery was widened with dissection of interlaced thin ligaments on the anterior surface of mesentery

Emergent laparoscopic operation was performed. The patient was placed in reverse Trendelenberg position. At a first laparoscopic glance, the cecum was located just under the liver at mid-abdomen (Fig. 3a). The small bowel showed a 180° clockwise volvulus, but with neither congestion nor ischemic signs. Using atraumatic bowel forceps, the small bowel was examined from distal ileum end to proximal with continuous spreading of the anterior mesentery surface in a stepwise fashion, ensuring no residual volvulus or local twists. After the complete volvulus reduction, the Ladd’s band (Fig. 3b) was divided (Fig. 3c) and the duodenum was mobilized with dissection of adhesions using SonoSurg™ ultrasonic surgical device (Olympus, Tokyo, Japan). For the separation of the duodenum and ileocecal region, mesentery was widened with dissection of interlaced thin ligaments on the anterior surface of mesentery. The gastrocolic ligament connecting Ladd’s band was additionally dissected to mobilize the right colon furthermore to the left (Fig. 3d). Appendectomy was added outside the umbilical porthole.

The postoperative course was uneventful, and the patient was discharged at the sixth postoperative day.

## Discussion

In the pediatric population, the presence of hepatic portal venous gas (PVG) has been described in association with various disease processes such as necrotizing enterocolitis, gastroenteritis, following umbilical catheter, bowel obstruction, Hirschsprung’s disease, duodenal stenosis, SMA syndrome, and hypertrophic pyloric stenosis [1, 8].

There are four main theories that explain the pathophysiology of PVG: (1) bacterial—intramural gas-forming bacterial proliferation, (2) mechanical—increased intraluminal pressure during gastric or intestinal obstruction, (3) mucosal damage—air enters through disrupted mucosa, and (4) pulmonary disease—alveolar air dissects down through the mediastinum to the gastric wall [1]. In some cases, these factors appear to contribute in combination [2].

Intramural gas bubbles detected as pneumatosis intestinalis (PI) may be absorbed into the intestinal venous system, may travel into the portal vein, and can be localized as PVG by real-time ultrasound as flowing echogenic dots. Finally, PVG is trapped in the small branches of the portal vein inside the liver inducing dense granular echogenicities in hepatic parenchyma [3].

Reports of PVG as well as PI detected in the case of intestinal malrotation are rare, and the most reported cases showed intestinal necrosis that necessitated bowel resection and showed poor prognosis [1, 4–7].

In the present case, the micro-bubbles of gas were detected as highly echogenic particles flowing within the intrahepatic portal vein, and this become an opportunity for further evaluation and the intestinal malrotation was diagnosed. Operative findings showed a mild volvulus with neither congestion nor ischemic change of the twisted bowel. Raised intraluminal pressure or direct stimulation of the bowel wall induced by the volvulus might allow gas to infiltrate the bowel wall and mesentery portal venous flow. Therefore, PVG in this case appeared to be in a transient process that resolves within a short interval after spontaneous winding down or decompression of twisted bowels.

Recent literatures have stated that midgut volvulus in malrotation can be managed well in infants without deteriorating condition. The laparoscopic approach is feasible and effective for the case that even shows PVG, and should have the advantage of minimally invasive surgery with small incisional scar.

## Conclusions

We reported a case of intestinal malrotation detected with air bubbles floating in the intrahepatic portal vein by ultrasonography. Transient portal venous gas bubbles may be generated even in the mild intestinal volvulus with no bowel ischemia. Sonography is very sensitive for PVG detection even with very small amounts of gas, and this sign could be one of the useful markers for detecting the early stage of volvulus.

## Data Availability

The authors declare that all data in this article are available within this published article.

## Abbreviations

CT: Computed tomography
PI: Pneumatosis intestinalis
PVG: Portal venous gas
